# Channel and Timeslot Co-Scheduling with Minimal Channel Switching for Data Aggregation in MWSNs

**DOI:** 10.3390/s17051030

**Published:** 2017-05-04

**Authors:** Sanggil Yeoum, Byungseok Kang, Jinkyu Lee, Hyunseung Choo

**Affiliations:** College of Software, Sungkyunkwan University, Suwon 16419, Korea; sanggil12@skku.edu (S.Y.); byungseok@skku.edu (B.K.); jinkyu.lee@skku.edu (J.L.)

**Keywords:** channel switching, channel and time slot co-scheduling, data aggregation

## Abstract

Collision-free transmission and efficient data transfer between nodes can be achieved through a set of channels in multichannel wireless sensor networks (MWSNs). While using multiple channels, we have to carefully consider channel interference, channel and time slot (resources) optimization, channel switching delay, and energy consumption. Since sensor nodes operate on low battery power, the energy consumed in channel switching becomes an important challenge. In this paper, we propose channel and time slot scheduling for minimal channel switching in MWSNs, while achieving efficient and collision-free transmission between nodes. The proposed scheme constructs a duty-cycled tree while reducing the amount of channel switching. As a next step, collision-free time slots are assigned to every node based on the minimal data collection delay. The experimental results demonstrate that the validity of our scheme reduces the amount of channel switching by 17.5%, reduces energy consumption for channel switching by 28%, and reduces the schedule length by 46%, as compared to the existing schemes.

## 1. Introduction

Recent advances in wireless sensor networks (WSNs) have enabled the development of low-cost and low-power devices that are smaller in size and have longer coverage distances. In the case of single-channel wireless sensor networks (SWSNs), nodes use identical channel information for both transmitting and receiving data. When two nodes communicate with each other, the surrounding nodes cannot transmit data until the communication between the two nodes ends. The multichannel mode was studied to overcome such a deficiency of the existing SWSNs. In MWSNs, the surrounding nodes can transmit data using different channels, where the pairing of transmitting and receiving nodes uses a specific channel. Due to the increase in the amount of simultaneously-transmitted data, the data collection rate can be improved in MWSNs. In addition, SWSNs consider only the time slot conflict, while MWSNs simultaneously consider both the channel and time slot conflict. As the channel and time slot are mutually independent resources, allocation/scheduling algorithms have to consider them separately.

Most existing studies on MWSNs consider time slot assignment, channel allocation, and switching. When neighbor nodes use different channels, the transmitting (sender) node transmits data by switching to the receiving channel of another node, and then returns to the receiving channel to receive data from other nodes. Channel switching requires calibrating and channel switching time. As the number of channel switches is increased, time synchronization problems occur [1]. Due to channel switching twice for one transmission from one node to another, a significant amount of energy and time are consumed. Since sensor nodes are energy-constrained, energy efficiency can be improved by reducing the energy consumed in channel switching.

Existing MWSN studies allocate different channels to all receiving nodes to prevent conflicts between channels, but time slot assignment is not taken into account. Assigning multiple channels to different nodes will require frequent channel switching at the nodes, resulting in decreased energy and time efficiency. In MWSNs, different channels and time slots can be used to complement each other in providing a collision-free schedule. This can be solved with different time slot assignments, since it does not require allocating multiple channels. Similarly, matters which require different channel assignment do not need to consider different time slots. However, minimal channels must be assigned to the nodes in order to reduce the switching count, energy consumption, and data aggregation delay.

This paper addresses the channel switching problem and proposes a scheme for efficient channel and time slot allocation for MWSNs. The proposed scheme allocates the same channel between neighbor nodes in order to reduce the number of channel switches using a set of multiple time slots in one period. Using the optimized time slot assignment along with the proposed channel allocation method, we saw a short data collection delay and no conflict between sensor nodes. In addition, the proposed scheme can reduce the amount of channel switching by using the same channel in one period with no conflict.

Contributions of the proposed scheme can be summarized as follows:

- Our key contribution is minimizing the number of channel switching. Channel switching cost is less than TX or RX. However, channel switching occurs at every TX and RX for data processing. We reduced the number of channel switches by channel and time slot co-scheduling.
- The channel allocation method of the proposed scheme reduces the amount of channel switching by taking into account the time slot assignment beforehand, according to variable duty.
- The time slot assignment scheme can transmit data to the sink node within the shortest time based on the bottom-up approach for the allocated channel.
- To evaluate the performance of the proposed scheme, network simulations are carried out in various environments. Simulation results show that up to 17.5% of channel switching is reduced which, in turn, reduces 28% of the energy consumption. With the increase in node density, the schedule length for data aggregation reduces by 46% in comparison to existing studies.

The rest of the paper is structured in the following manner: Section 2 describes related studies; Section 3 defines problems with the network model, and assumptions are discussed; the proposed scheme for channel and time slot assignment in a multichannel wireless sensor network is thoroughly explained in Section 4; Section 5 presents the simulation environment and performance evaluation of the proposed scheme; and, finally, Section 6 concludes the work.

## 2. Related Work

Multichannel multiple access control (MAC) protocols have been widely researched in sensor networks, which can be categorized according to the sensor mote environment and channel assumptions. Some studies focused on channel selection by scanning when a channel change request arrives [2,3,4,5,6,7]. However, each sensor device cannot transmit and receive data simultaneously on different channels because a single transceiver (hardware module) is installed at each sensor device, and one device cannot use several channels simultaneously. A different method to listen on only selected channels is researched by [8,9,10]. In their works, each sensor receives data through a receiving channel, and transmits data by switching to the target channel of the corresponding node. After that, it returns to its receiving channel. This protocol reduces the energy consumption in comparison to the protocols which are listening to all channels. In this paper, we transmit and receive data by listening only to allocated channels.

The tree-based multichannel allocation method is another solution [11,12,13]. The tree-based multi-channel protocol (TMCP) connects nodes without interference between trees. It uses a distance-based interference model and allocates channels [14]. This method still results in channel conflict; however, it is partially solved by re-transmitting failed data. Concerning the RSS-based multichannel allocation method, channels are allocated via the highest intensity of a single node [15,16]. By using this technique, a node can communicate to its neighbor with minimal interference, but channel interference still exists.

An interference-free method has been widely studied [17,18,19,20,21]. This method allocates different channels to remove the channel interference for neighbors. It is defined as an NP-hard problem. The channels are automatically allocated by calculating the transmission rate through a heuristic method [18]. Sensing data can be transmitted and received without channel interference of the nodes, but the number of channels significantly increases. If the node density is increased, calculation of channel allocation is required.

Several studies have discussed collection with minimum transmission delay [22,23,24,25,26,27,28]. These studies aim at minimizing time slot conflict and increasing the number of transmitting nodes without the interference of channels. Through bottom-up approaches (time slot and channel allocation), the schedule length for data aggregation is reduced in [22]. To solve the minimum data aggregation time problem, subset-based interference-free time slot allocation is proposed in [23]. The paper proposed assigning time slots with a minimum schedule length scheme based on the previous interference-free channel allocation.

The largest degree first (LDF) algorithm is presented to prevent channel conflicts [24]. A channel conflict for data transmission is prevented by allocating channels to the nodes in the order of the highest degree of the constraint graph. Similarly, time slot conflict is resolved by assigning different time slots to the surrounding nodes. The performance of the receiver-based constraint graph (RCG) and link-based constraint graph (LCG) are compared in [29]. These schemes require additional Δ+1 channels. This is a main problem with RCG and LCG. For that reason, we focused on reducing the number of channels and schedule length without conflict by using RCG.

The energy consumption and delay for channel switching is studied in the multi-channel sensor model [1,30,31]. They measured different values in the MicaZ mote in the Chipcon CC1000 radio environment. Through extensive experiments, they found that, for the Mica2 nodes, (1) there are 48 channels available; (2) it requires about 50 ms to switch between two channels; and (3) it consumes about 1940 nJ of energy to switch between two channels. They also point out some implications of these results for the design of multi-channel protocols.

## 3. Preliminaries

### 3.1. Network Model and Assumption

The network model used in this paper consists of a sensor network with a number of sensor nodes and one sink node to perform data aggregation. We assume that each sensor node has an omnidirectional antenna, and the same transmission range. The basic network topology is expressed as a non-directional graph, G=(V,E), where V consists of of all the sensor nodes within the network, and E represents all of the wireless links between the sensor nodes. If node v is in the communication range of node u, the two nodes have a link and can communicate with each other. Every sensor node has at least one path to the sink node through neighbor nodes within its communication range.

In our simulation, the sensor nodes have multiple channels and active time slots. At the first time (node deployment), time slots and channels are not allocated to the sensor nodes. This means that they are allocated later based on the basic policy of the scheme. If a node wants to transmit data to its neighbor, it changes its channel to the same one as the neighbor. After that, this node returns back to the original channel for receiving data from another neighbor. A single node consists of several periods; the length of each period is *L* and has the same number of active slots. Other time slots (not the active time slot) are in the sleep state, and the duty period is set as 1/*L*. The problem of transmission and receiving conflicts is resolved by assigning different channels and time slots. However, if the channel and the active time slot have the same number, a conflict occurs. This problem (secondary conflict) can be solved by using multiple channels. Each node can transmit or receive sensing data at a time upon data transmission (half duplex). Furthermore, a sensor node can transmit data in any time slot, but data receiving is possible only in the allocated active time slot.

As we mentioned previously, transmitting and receiving nodes can communicate in the allocated channels and time slots, but they cannot communicate simultaneously. The conflicts that can occur in such an environment are categorized into primary and secondary conflicts. The primary conflict occurs when one node receives data from two or more nodes. The secondary conflict occurs at a node through unintended interference by another ongoing transmission while the node is receiving data from a neighbor node. In the multi-channel sensor network environment, two receiving nodes having the same channel and time slot is a main reason for secondary conflict.

The RCG is used to prevent channel conflict [24]. The RCG is created by a combination of the network graph G=(V,E) and mainly consists of the receiving nodes in *G*. In the RCG $G_{C}=\left(V_{C},\;E_{C}\right)$, $V_{C}$ is the set of receiving nodes (include the sink), and $E_{C}$ is the set of the edges connecting two nodes where secondary conflict occurs. For the two nodes u and v in the graph, when one or more neighboring nodes of v (likewise u) are from the descendants of u (likewise v), secondary conflict may occur between nodes u and v.

### 3.2. Problem Formulation

In the receiving node-based channel allocation schemes, each node obtains a fixed channel to receive the sensing data from its neighbor node. With this context, previous studies considered channel conflicts only upon channel allocation. Actually, conflicts occur in all channels and time slots, but they have no relationship for mutual conflicts since they are composed of independent resources.

#### 3.2.1. Problem 1: Channel Switching Delay and Energy Consumption

In previous studies of RCG, they allocate different channels to avoid channel conflict for the neighbors. This requires frequent channel switching for every transmission or reception. In this case, the required number of allocated channels is Δ+1. As we have shown, energy consumption and delay may occur for channel switching [19]. For instance, in MicaZ, data transmitting, data receiving, and channel switching for 1024 bits of data consumes 212.9, 230.4, and 1.94 μJ, respectively. The energy consumption of channel switching is much smaller than those of transmitting and receiving. However, if the sender and receiver have different receiving channels for every transmission, it is not negligible. If the same channel is used between neighbors, channel switching does not occur. We can reduce the energy consumption by using this strategy. This paper focuses on reducing the energy cost of the channel switching by allocating different available time slots.

#### 3.2.2. Problem 2: Channel Allocation and Time Slot Assignment

Previous studies solved channel conflict by allocating different channels and time slots separately. However, conflict does not occur if we use the strategy of either different time slot assignment or different channel allocation. Additionally, the amount of channel switching and the required number of channels are decreased if the same channel is allocated to two neighbor nodes.

**Figure 1:**
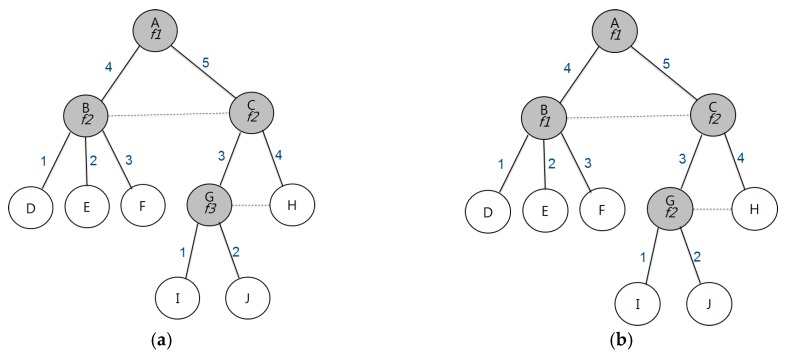
Comparing the channel optimization in the same tree: (**a**) using three channels; and (**b**) using two channels.

Figure 1a shows channel allocation using the RCG algorithm. In this figure, there are three channels that are allocated, and channel switching is required for every transmitting and receiving phase. Figure 1b shows the allocation of different time slots between random nodes. To avoid channel conflict, we allocate the same channel between transmitting and receiving nodes. In this figure, we use two different channels (*f*1 and *f*2). No channel switches occurs when data is sent from B to A, and from G to C. Channel switching occurs only when data is sent from C to A.

## 4. Channel and Time Slot Co-Scheduling for Minimal Channel Switching

The proposed scheme is divided into a channel allocation stage that minimizes channel switching and the time slot allocation stage for data aggregation with minimal delay. This chapter describes the basic mechanism and operational procedure of the proposed scheme. Table 1 shows the notation used in this chapter.

### 4.1. Channel Allocation

**Figure 2:**
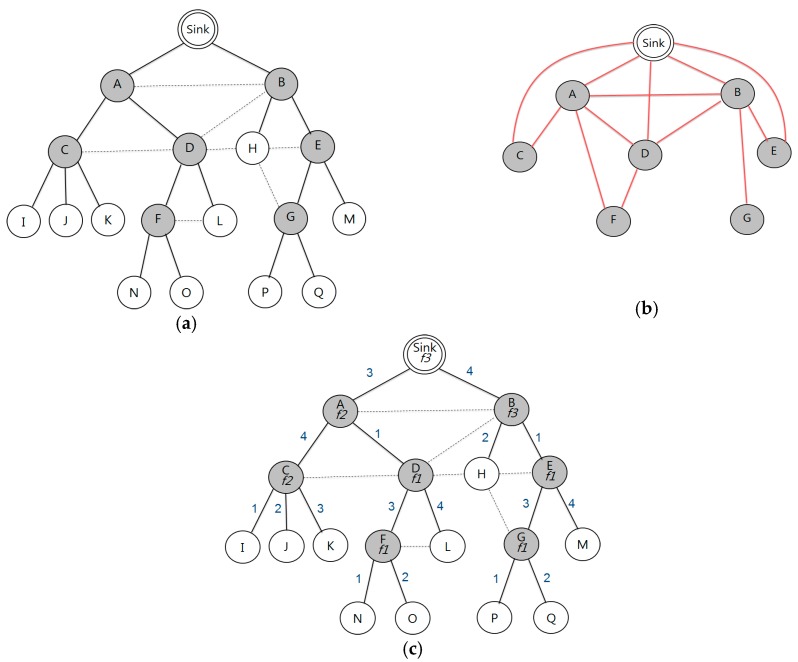
Example: (**a**) communication graph; (**b**) receiver-based constraint graph; and (**c**) minimal switching graph.

The proposed channel allocation scheme reduces the amount of channel switching by assigning the same channel to neighboring nodes, but different time slots, in order to avoid conflict. Conflict between channels is prevented by using the RCG. Algorithm 1 shows the mechanism of the RCG. For each node in *Vc*, the node finds a secondary conflict from its neighbor and child nodes. If there are nodes that can experience channel conflict, then we connect each of them for conflict-free scheduling. The result of Algorithm 1 is illustrated in Figure 2b.

Figure 2a shows the communication graph. A dotted line means interference between the nodes, and receiver nodes are shaded. In Figure 2, if the value of L is 3, channel allocation starts from node C, whose |u.descendent (node C)| is 3, and if the value of L is 10, channel allocation starts from node A, whose |u.descendent (node A)| is 9. This means that node C can receive data from its descendant nodes—I, J, and K—within one period, when L is 3 or higher. The number of channel switching is reduced by allocating the same channel to the non-conflict nodes. In addition, we can avoid the conflicts through time slot division.

If a child node has one or more receiving nodes, the same channel is assigned to both the child and parent nodes. The subtree is constructed of a single period of a time slot, and channel conflict does not occur because of the assignment of a different time slot in the subtree. If a subtree has multiple numbers of receiving nodes, channel allocation begins from the nodes with the lower ID. In Figure 2c, the same channel is allocated to both nodes D and F, because node F is a child of node D, when L is 4. Therefore, nodes F, L, N, and O can transmit data to node D in a period without channel conflicts, and the same channel is allocated to the nodes D and F. After finishing the channel allocation in the subtree, the next subtree is formed for channel allocation in the number of remaining nodes. Continuing this way, channels are allocated sequentially from the bottom node (child) to the sink (parent) in the tree.

**Algorithm 1.** Constraint Graph Construction**Input:** G=(V,E),$\;T=\left(V,\;E_{T}\right)$

**Output:** Constraint graph $G_{C}=\left(V_{C},\;E_{C}\right)$
    /* Constraint Graph Construction */
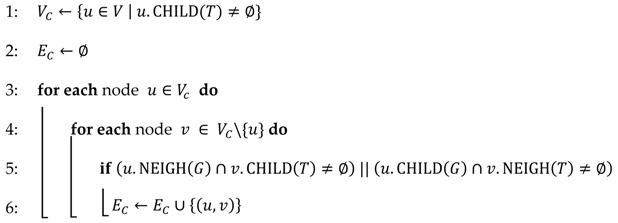


In Figure 2, when channels are allocated to nodes D and F, the subtree size of node A becomes 5 and the subtree size of the sink becomes 13, respectively. This causes no data to be received in a single period, as five nodes remain for channel allocation under the three of node A. In the case of node E, where the subtree size is 4 and lower, channels are allocated using the same method. The channels of nodes D and F are already allocated; we can check for channel conflict among them using the RCG. In Figure 2b, channel conflict does not occur between nodes D and F; E and G, although the same channel is allocated to nodes D, E, F, and G, because no edge connects nodes F and G. If there exists a connection between nodes F and G in the RCG, they can avoid channel conflict by assigning different channels. Table 2 shows the subtree size according to channel allocation. 

### 4.2. Time Slot Assignment

In the proposed scheme, time slot assignment uses the same subtrees which were previously established in the stage of channel allocation. Initially, time slots are allocated to the links in a subtree, followed by the time slot assignment to the link between the subtrees. We use a sequential bottom-up approach for time slot assignment in a tree. This approach shows higher performance compared with the traditional scheme.

In our scheme, the nodes in a subtree can transmit data without any conflict while using different time slots in a period. Since channels are also allocated within a subtree, data can be collected by a subtree root within one period. To prevent the conflicts, different time slots are always assigned to the nodes that are allocated the same channel to reduce channel switching.

It is necessary to assign a time slot to link between two subtrees based on the completion of the time slot assignment in a subtree. To assign a time slot to a link between two subtrees may cause primary conflict in the bottom-up approach when the time slot is already being used in one of the trees for data transmission. We can avoid such conflict by using the least-valued time slot not used in the subtree.

Figure 2c shows time slot assignment through the bottom-up approach in a subtree. Time slot 1 is assigned to the link between A and D where two subtrees are connected. node A uses time slot 3 for data transmission to the sink, and uses time slot 4 to receive data from node C. In the same manner, node D uses time slots 3 and 4 to receive data from nodes F and L. Between the remaining time slots 1 and 2, the fastest—time slot 1—is assigned for node D to receive data from node A.

**Figure 3:**
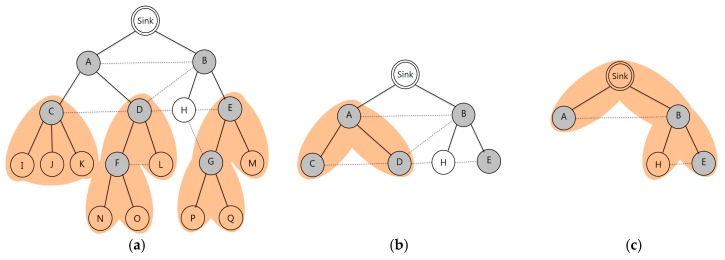
Example: channel assignment sequence by subtree: (**a**) a channel assigned to nodes C, D, E, F, and G; (**b**) a channel assigned to node A; and (**c**) a channel assigned to node B and the sink.

Algorithm 2 shows the mechanism of the channels and time slot scheduling in the proposed scheme. *R* is the set of nodes that have their descendent nodes that are less than, or equal to, the length of the time slot in a period. Set *I* is the set of the used channels from neighbor nodes in the RCG. We can avoid channel interference by using this set. In this scheme, time slots start from 1 to *L*. Sets *R* and *I* are changed through the operation of remove and update. The details of this cycle are illustrated in Figure 3.

**Algorithm 2.** Channel and Timeslot Co-Scheduling**Input:**
$T=\left(V,E_{T}\right),\;\;G_{C}=\left(V_{C},E_{C}\right)$, *L*, $S_{CH}$
    // *L*: length of time slot in a period, $S_{CH}$: set of given channels 
**Output:** Channel and timeslot assignment for every node u∈V
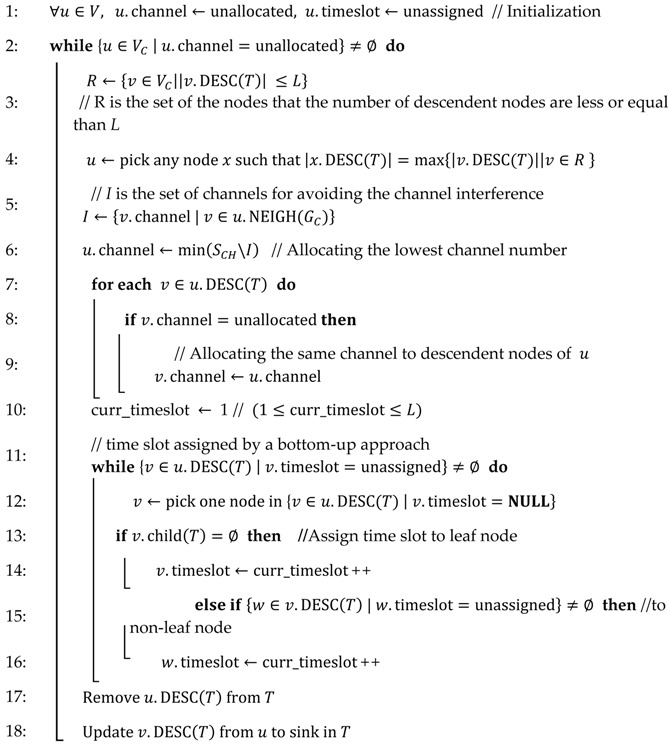


### 4.3. Comparison

**Figure 4:**
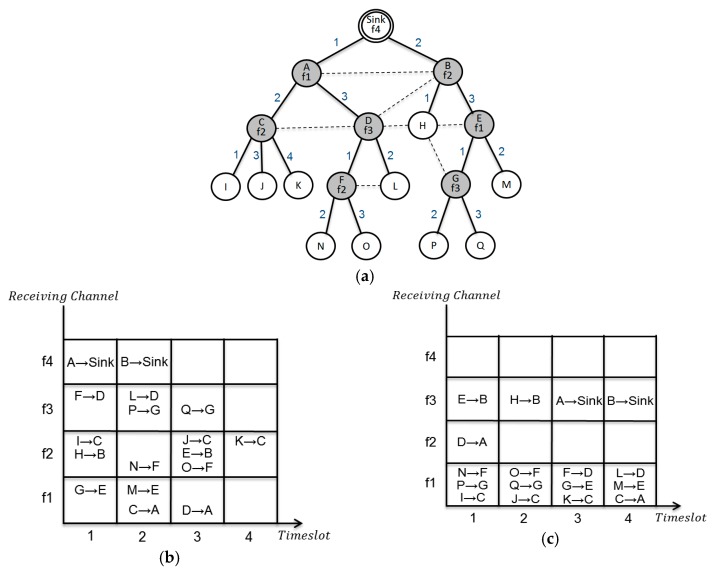
Example: channel and time slot scheduling between the LDF and the proposed scheme: (**a**) channel and time slot scheduling by the LDF; (**b**) channel and time slot scheduling table of Figure 4a; and (**c**) channel and time slot scheduling table of Figure 2c.

The existing RCG based schemes allocate different channels to all of the receiving nodes. Figure 4a shows the channel allocation based on the previous scheme described in Figure 2a. When data is sent from node G to the sink node, nodes B, E, and G should switch channels to transmit data to the upper level node. After that, they return back to their own allocated channel to receive the data from the lower nodes. In Figure 4a, total of 14 channel switches are required for all data to be gathered at the sink. However, our scheme can reduce the number of channel switches. In our case, channel switching occurs only in node E, and for nodes B and G no channel switching is required. This scheme requires only eight channel switches, as no channel switching occurs with nodes B, F, and G. Figure 4b,c shows the channel allocation to all of the receiving nodes and shows a definite decrease in the number of allocated channels for a conflict-free data transmission, respectively.

## 5. Performance Evaluation

### 5.1. Simulation Environment

In this section, we discuss the results of the performance evaluation through network simulation in various scenarios. Environmental settings for the simulation are as follows: the network graph uses the unit disk graph (UDG), and the number of nodes is fixed at 100. Sensor nodes are placed randomly in the 200 × 200 m area of a two-dimensional space. We assumed the sensing data was collected before transmitting to their parent node. The nodes can transmit all data at once and the receiver node also allows it. We repeat the simulation 400 times for each combination of the following two parameters:

- Transmission range: 20, 25, 30, 35, 40, 45, 50 m
- The length of a period (*L*): 4 (default), 6, 8, 10 (duty: 25, 16.6, 12.5, and 10%)

Every node has the same duty and periodically repeats the wake-up and sleep mode following the duty. MicaZ is used for the energy model of the simulation. The initial energy of the node is 2 J, and the data packet size is fixed to 1024 bits. For one-time data transmission with 208 nJ/bit, 208 nJ × 1024 bits = 212.9 μJ is consumed. For data receiving with 225 nJ/bit, 225 nJ × 1024 bits = 230.4 μJ is consumed in one receiving phase. Channel switching consumes 1.94 μJ. Idle listening (56.4 mW) and sleep (3 μW) costs are one of the big parts of energy consumption in real-world WSNs. However, it is not our concern, because all nodes in both networks consumed the same amount of energy in idle listening and sleep states. Furthermore, these fundamental costs do not make any change in experimental result.

### 5.2. Number of Channels

**Figure 5:**
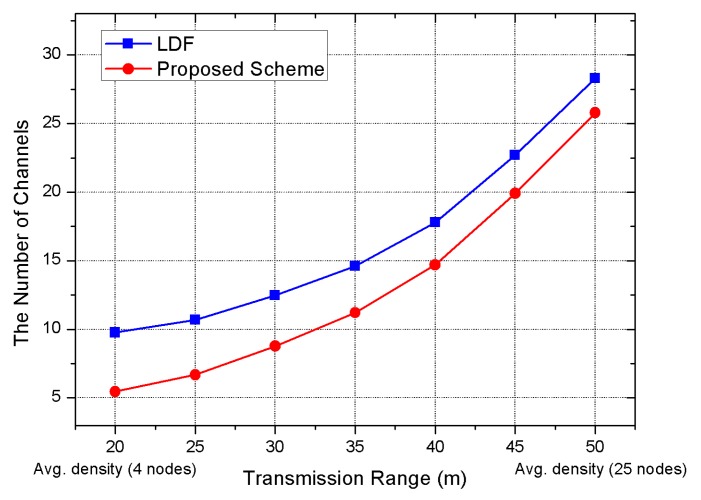
Simulation result on the number of channels used according to the transmission range; Avg. node density from 4 (20 m) to 25 (50 m).

Firstly, we measured the number of channels while increasing the transmission range of a node. If the transmission range increases, a large amount of interference occurs. As a simulation result, the proposed scheme shows a smaller number of required channels compared with the LDF scheme. If there are more neighboring nodes, the efficiency of the proposed channel allocation scheme is reduced. However, the proposed scheme shows better performance than the LDF. The reason is that the number of links connected with the RCG increases, as there are more neighboring nodes and, thus, the probability of using different channels for surrounding neighboring nodes becomes higher. The LDF needs 27.8 channels when the transmission range is 50 m, and the proposed scheme uses 25.7 channels, implying that 7.5% of channel reduction is shown in Figure 5. When the transmission range is 25 m, the previous scheme needs 11.3 channels, while the proposed scheme uses 6.8 channels, which exhibits 40% channel reduction. As the transmission range decreases, the efficiency of the proposed scheme increases.

### 5.3. Channel Switching

**Figure 6:**
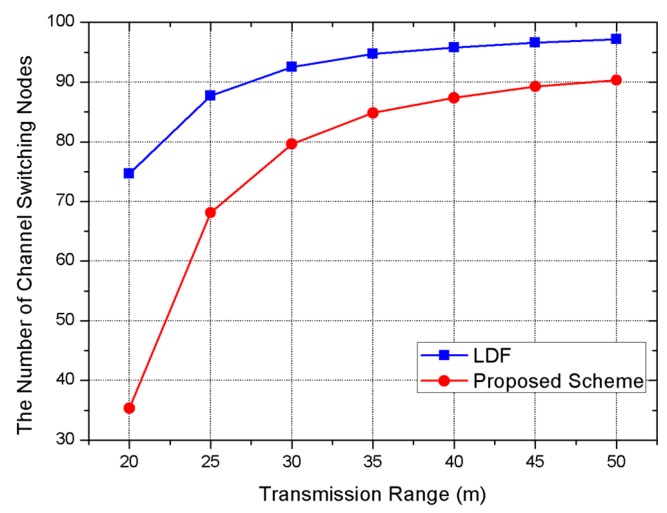
Simulation result on the number of channel switching nodes.

In Figure 6, when the transmission range is 25 m, 88.1 nodes need channel switching in the LDF, while 69.7 nodes need channel switching in our scheme. The main reason is that the number of idle nodes is increased as the number of neighboring nodes decreases. The transmission range is not affected when all of the different channels are allocated to neighboring nodes because different channels are allocated to all nodes. However, the efficiency of the proposed scheme declines gradually when the number of neighboring nodes increases.

**Figure 7:**
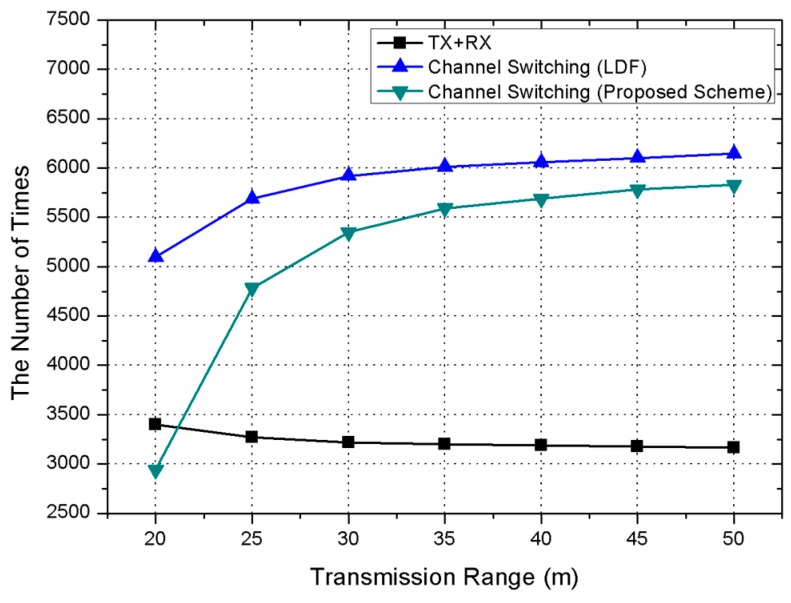
Simulation result on the number of switching and communication times.

Figure 7 shows the measurement of the number of transmissions and receptions, and the amount of channel switching according to the transmission range changes. The proposed scheme reduces the energy consumption for switching compared to the previous scheme. This simulation uses the same number of transmissions and receptions for comparison with the previous scheme. If the transmission range is 25 m, the number of transmissions and receptions is 3300, and the LDF needs to switch channels 5700 times. However, the proposed scheme needs to switch channels 4750 times. As the transmission range increases, the amount of switching in the proposed scheme increases. This means that using different channels between neighboring nodes goes up frequently, due to the increase of interference.

**Figure 8:**
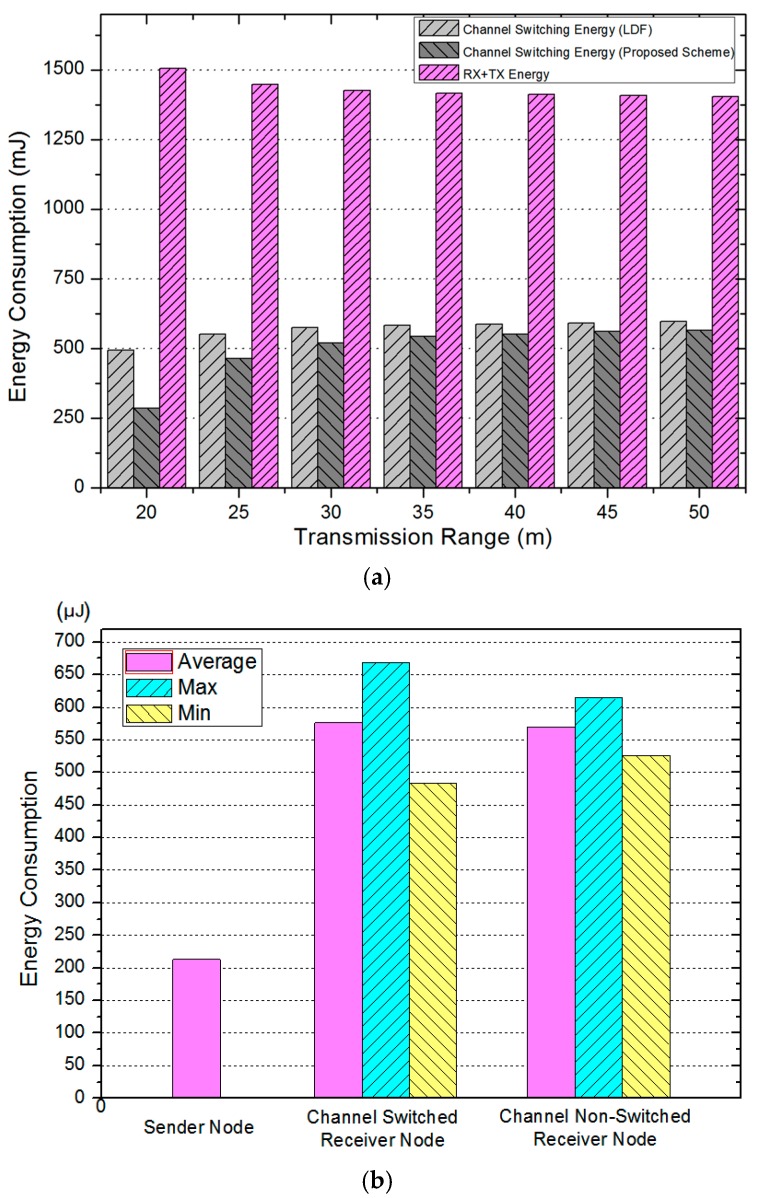

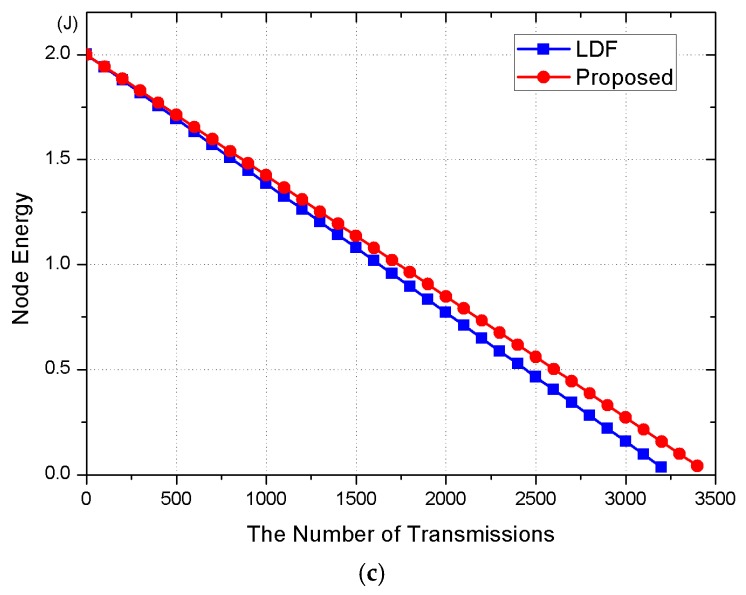
(**a**) Energy consumption simulation results; (**b**) energy consumption by node role; and (**c**) remaining node energy according to the number of transmission.

Figure 8a compares the energy consumption for channel switching when the number of transmissions and receptions is the same. When 2 J energy is consumed, the previous scheme consumes 550 mJ of energy for channel switching when the transmission range is 25 m, while the proposed scheme consumes 400 mJ energy. The proposed scheme can transmit and receive data more than the previous scheme. Figure 8b shows the energy consumption required for one-time data collection based on the roles of the node. The maximum and minimum indicate the energy consumption values of the node using the maximum and minimum energy in the topology. The deviation of the maximum/minimum is larger in the channel switching nodes rather than the non-switching nodes. The reason is that the amount of transmitting and receiving data is smaller than the number of channel switching nodes. The possibility of selecting an idle node is higher since the number of descendant nodes is smaller. Figure 8c compares the remaining energy of the node according to the number of transmissions until consuming 2 J. Figure 8c indicates the mean (average) value of the receiving nodes until energy becomes zero (death node). In the LDF, we can see the zero energy node after 3200 transmissions. In our scheme, the nodes in which energy becomes zero are generated after 3450 transmissions.

### 5.4. Schedule Length

**Figure 9:**
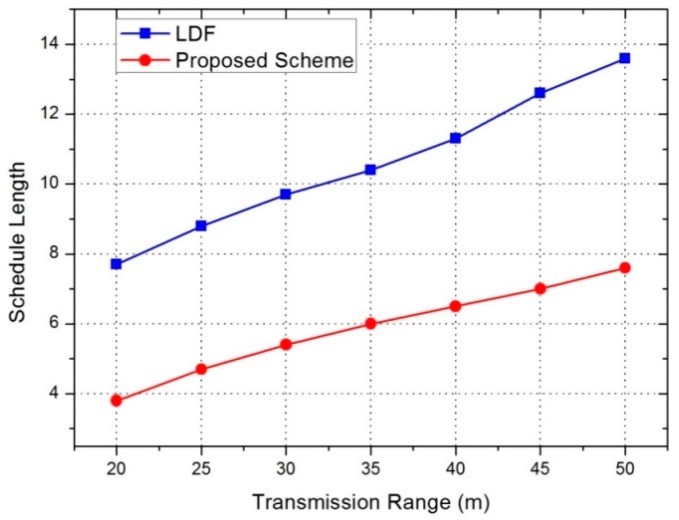
Simulation result on schedule length.

Schedule length indicates the number of periods used until the sink node receives data from all senders. Schedule length is tightly related with the data collection rate, and its lower value means a higher data collection rate (throughput). In Figure 9, when the transmission range is 25 m, the LDF scheme needs 8.8 periods, but the proposed scheme needs 4.8 periods. Therefore, a 46% performance improvement can be seen with the time slot assignment method in the proposed scheme. As the transmission range increases, the performance of the proposed scheme increases. This means that the efficiency of the bottom-up approach reduces delays by minimizing the schedule length.

### 5.5. Impact on the Duty Period

**Figure 10:**
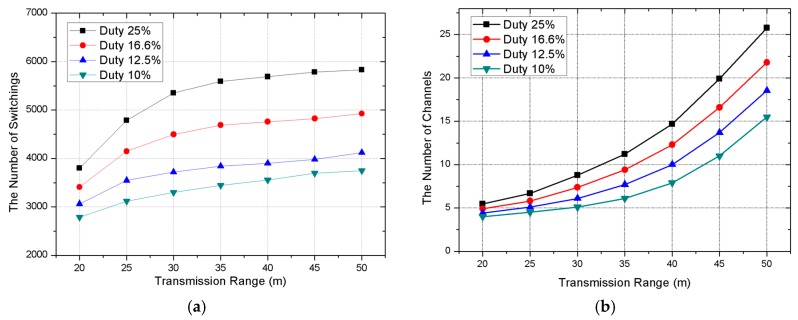
(**a**) The number of channels required according to the duty period; and (**b**) the number of channel switches according to the duty period.

The number of time slots in one period is decided according to duty. If the number of time slots increases, the duty is lower. In our scheme, we have more chance to allocate the same channels between neighbor nodes as we increase the number of time slots in one period. Figure 10 shows the decrease in the number of channels with a low value of duty. As the transmission range increases, the number of neighbor nodes increases and, thus, the number of required channels rapidly increases.

**Figure 11:**
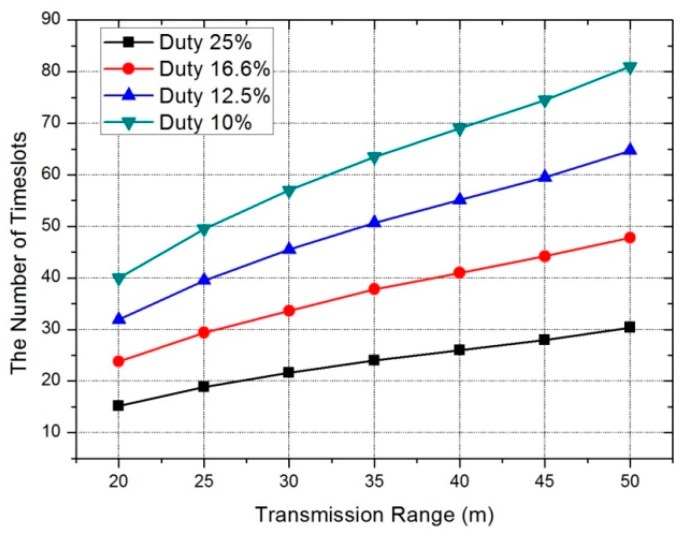
Schedule length according to duty period.

The number of time slots in one period changes according to duty, as does the number of time slots required for all nodes to transmit data to the sink node. Schedule length is related with the time slot assignment. As seen in Figure 11, if the time slot length in one period increases (due to low duty), the schedule length becomes longer. Although the schedule length becomes longer (duty becomes lower), the amount of channel switching is reduced. For that reason, the optimized duty setup is necessary according to the constitution of the topology.

## 6. Conclusions

This paper proposed a method to allocate channels and assign time slots to reduce channel switching and minimize data collection delays in a multichannel environment. Previous schemes (LDF) avoided channel conflict by using different channels between neighbors without consideration of channel switching overhead. However, this approach is not appropriate in multichannel WSN. In this paper, we minimized channel switching by allocating the same channel between neighbors after calculating the size of the subtree. The proposed scheme also minimized data collection delays through the bottom-up mode time slot assignment. Through the network simulation, the proposed scheme improved the network lifetime. In addition, we minimized the energy consumption by reducing the number of channels and the amount of channel switching. As a result, the number of switches, the energy required for channel switching, and the schedule length was reduced by 17, 28, and 46%, respectively.

There are two limitations in this paper. First, the node degree of the communication tree has a restriction. It should be less than the number of timeslots in a period (less than *L*). Second, this scheme cannot apply to distributed WSNs. The sink should know the connectivity of nodes and its interferences. We have a plan to improve the proposed scheme as a distribution mode to be suitable for the more realistic multichannel WSNs. Furthermore, we will extend the previous RCG scheme to reduce the schedule length and enhance the channel allocation efficiency.

## Figures and Tables

**Table 1:** List of notations.

G=(V, E)	Communication Graph of a Network V: Set of Nodes, E: Set of Edges
$G_{C}=\left(V_{C},\;E_{C}\right)$	Constrant graph of a network
$T=\left(V,\;E_{T}\right)$	Communication tree from *G*; *E_T_*: set of edges in the tree
*L*	The number of time slots in a period
u. channel	Allocated channel of Node u
u.timeslot	Assigned time slot of Node u
*u*.CHILD	The set of child nodes of Node u
*u*. DESC	The set of descendent nodes of Node u
u. NEIGH	The set of neighbor nodes of Node *u*

**Table 2:** The number of descendant nodes to which channels are not allocated according to channel allocation.

Node ID	Unassigned Descendent Nodes	Channel Assigned to Nodes D and F	Channel Assigned to Nodes E and G	Channel Assigned to Node C	Channel Assigned to Node A	Channel Assigned to Nodes B and Sink
Sink	17	13 (=17 − 4)	9 (=13 − 4)	6 (=9 − 3)	4 (=6 − 2)	-
A	9	5 (=9 − 4)	5	2 (=5 − 3)	-	-
B	6	6	2 (=6 − 4)	2	2	-
C	3	3	3	-	-	-
D	4	-	-	-	-	-
E	4	4	-	-	-	-
F	2	-	-	-	-	-
G	2	2	-	-	-	-

## References

[1] Chen H., Cui L., Lu S. An experimental study of the multiple channels and channel switching in wireless sensor networks. Proceedings of the 4th International Symposium on Innovations and Real-Time Applications of Distributed Sensor Networks.

[2] Zhou G., Huang C., Yan T., He T., Stankovic J.A., bdelzaher T.F. MMSN: Multi-Frequency Media Access Control for Wireless Sensor Networks. Proceedings of the 25th IEEE International Conferences on Computer Communications.

[3] So J., Vaidya N.H. Multi-channel MAC for ad hoc networks: Handling multi-channel hidden terminals using a single transceiver. Proceedings of the 5th ACM International Symposium on Mobile ad Hoc Networking and Computing.

[4] Li J., Haas Z.J., Sheng M., Chen Y. Performance Evaluation of Modified IEEE 802.11 MAC for Multi-Channel Multi-Hop Ad Hoc Network. Proceedings of the 17th International Conference on Advanced Information Networking and ApplicationsApplications.

[5] Nasipuri A., Zhuang J., Das S.R. A Multichannel CSMA MAC Protocol for Multihop wireless Networks. Proceedings of the IEEE Wireless Communications and Networking Conference.

[6] Chen X., Han P., He Q., Tu S., Chen Z. A Multi-Channel MAC Protocol for Wireless Sensor Networks. Proceedings of the Sixth IEEE International Conference on Computer and Information Technology.

[7] Zhuo S., Wang Z., Song Y., Wang Z., Almeida L. (2016). A traffic adaptive multi-channel MAC protocol with dynamic slot allocation for WSNs. IEEE Trans. Mob. Comput..

[8] Zhang J., Zhou G., Huang C., Son S., Stankovic J.A. TMMAC: An Energy Efficient Multi-Channel MAC Protocol for Ad Hoc Networks. Proceedings of the IEEE International Conference on Communications.

[9] Wu S., Lin C., Tseng Y., Sheu J. A New Multi-Channel MAC Protocol with On-Demand Channel Assignment for Multi-Hop Mobile Ad Hoc Networks. Proceedings of the International Symposium on Parallel Architectures, Algorithms and Networks.

[10] Ren J., Zhang Y., Zhang N., Zhang D., Shen X. (2016). Dynamic Channel Access to Improve Energy Efficiency in Cognitive Radio Sensor Networks. IEEE Trans. Wirel. Commun..

[11] Soua R., Minet P. A Survey on Multichannel Assignment Protocols in Wireless Sensor Networks. Proceedings of the 2011 IFIP Wireless Days (WD).

[12] Adbulaziz M., Simon R. Multi-Channel Network Coding in Tree-Based Wireless Sensor Networks. Proceedings of the International Conference on Computing, Networking and Communications (ICNC).

[13] Yigit M., Incel O.D., Gungor V.C. (2014). On the interdependency between multi-channel scheduling and tree-based routing for WSNs in smart grid environments. Comput. Netw..

[14] Wu Y., Stankovic J.A., He T., Lin S. Realistic and Efficient Multi-Channel Communications in Wireless Sensor Networks. Proceedings of the IEEE 27th Conference on Computer Communications.

[15] Torregoza J.P.M., Hwang W. Multi-channel Multi-Transceiver Routing Protocol for Wireless Mesh Network. Proceedings of the 9th International Conference on Advanced Communication Technology.

[16] Nasipuri A., Das S.R. Multichannel CSMA with signal power-based channel selection for multihop wireless networks. Proceedings of the IEEE Vehicular Technology Conference.

[17] Tzamaloukas A., Garcia-Luna-Aceves J.J. A Receiver-Initiated Collision-Avoidance Protocol for Multi-Channel Networks. Proceedings of the Twentieth Annual Joint Conference of the IEEE Computer and Communications Societies.

[18] Xu W., Trappe W., Zhang Y. Channel surfing: Defending wireless sensor networks from interference. Proceedings of the 6th International Symposium on Information Processing in Sensor Networks.

[19] Xing G., Sha M., Huang J., Zhou G., Wang X., Liu S. Multi-Channel Interference Measurement and Modeling in Low-Power Wireless Networks. Proceedings of the 30th IEEE Real-Time Systems Symposium.

[20] Bagaa M., Younis M., Ksentini A., Badache N. (2014). Reliable multi-channel scheduling for timely dissemination of aggregated data in wireless sensor networks. J. Netw. Comput. Appl..

[21] Wu Y., Liu K.S., Stankovic J.A., He T., Lin S. (2016). Efficient Multichannel Communications in Wireless Sensor Networks. ACM Trans. Sens. Netw..

[22] Xiao S., Li B., Yuan X. (2015). Maximizing precision for energy-efficient data aggregation in wireless sensor networks with lossy links. Ad Hoc Netw..

[23] Ji S, He J.S., Cai Z. (2014). Data gathering in wireless sensor networks. Art Wirel. Sens. Netw..

[24] Ghosh A., Incel O.D., Kumar V.S.A., Krishnamachari B. (2011). Multichannel Scheduling and Spanning Trees: Throughput-Delay Tradeoff for Fast Data Collection in Sensor Networks. IEEE/ACM Trans. Netw..

[25] Chen X., Hu X., Zhu J. (2005). Improved algorithm for minimum data aggregation time problem in wireless sensor networks. J. Syst. Sci. Complex..

[26] Luo S., Yongmei S., Yuefen J. (2015). Data collection for time-critical applications in the low-duty-cycle wireless sensor networks. Int. J. Distrib. Sens. Netw..

[27] Nordin N., Clegg R.G., Rio M. Multichannel Cross-Layer Routing for Sensor Networks. Proceedings of the 23rd International Conference on Telecommunications (ICT).

[28] Zhang H., Soldati P., Johansson M. (2013). Performance bounds and latency-optimal scheduling for convergecast in WirelessHART networks. IEEE Trans. Wirel. Commun..

[29] Ma J., Wei L., Li X.-Y. (2014). Contiguous link scheduling for data aggregation in wireless sensor networks. IEEE Trans. Parallel Distrib. Syst..

[30] Agarwal S., De S. (2016). Dynamic spectrum access for energy-constrained CR: Single channel versus switched multichannel. IET Commun..

[31] Wu W., Luo J., Yang M., Li X. Energy Efficient Channel Assignment with Switching Optimization in Multi-radio Wireless Networks. Proceedings of the IEEE International Conference on Systems, Man, and Cybernetics.

